# Spatial distribution of the chromosomal forms of *anopheles gambiae *in Mali

**DOI:** 10.1186/1475-2875-7-205

**Published:** 2008-10-10

**Authors:** Nafomon Sogoba, Penelope Vounatsou, Magaran M Bagayoko, Seydou Doumbia, Guimogo Dolo, Laura Gosoniu, Sékou F Traoré, Thomas A Smith, Yéya T Touré

**Affiliations:** 1 Malaria Research and Training Center, Faculté de Médecine, de Pharmacie et d'Ondoto-Stomatologie, Université de Bamako, BP. 1805, Bamako, Mali; 2 Department of Public Health and Epidemiology, Swiss Tropical Institute, P.O. Box, CH-4002, Basel, Switzerland; 3 Vector Biology and Control, WHO Regional Office for Africa WR/Gabon, PO Box 820, Libreville, Gabon; 4 Special programme for Research and Training in Tropical Diseases (TDR), World Health Organization, CH-1211 Geneva, Switzerland

## Abstract

**Background:**

Maps of the distribution of malaria vectors are useful tools for stratification of malaria risk and for selective vector control strategies. Although the distribution of members of the *Anopheles gambiae *complex is well documented in Africa, a continuous map of the spatial distribution of the chromosomal forms of *An. gambiae s.s. *is not yet available at country level to support control efforts.

**Methods:**

Bayesian geostatistical methods were used to produce continuous maps of the spatial distribution of the chromosomal forms of *An. gambiae s.s*. (Mopti, Bamako, Savanna and their hybrids/recombinants) based on their relative frequencies in relation to climatic and environmental factors in Mali.

**Results:**

The maps clearly show that each chromosomal form favours a particular defined eco-climatic zone. The Mopti form prefers the dryer northern Savanna and Sahel and the flooded/irrigated areas of the inner delta of the Niger River. The Savanna form favours the Sudan savanna areas, particularly the South and South-Eastern parts of the country (Kayes and Sikasso regions). The Bamako form has a strong preference for specific environmental conditions and it is confined to the Sudan savanna areas around urban Bamako and the Western part of Sikasso region. The hybrids/recombinants favour the Western part of the country (Kayes region) bordering the Republic of Guinea Conakry.

**Conclusion:**

The maps provide valuable information for selective vector control in Mali (insecticide resistance management) and may serve as a decision support tool for the basis for future malaria control strategies including genetically manipulated mosquitoes.

## Background

Malaria remains one of the main public health problems in Africa and researchers are developing new vector control methods focused on the genetic manipulation of mosquitoes. The principles of the genetic control methods are based on the propagation of sterility or other desirable genetic factors in successive generations of mosquitoes [1,2]. The most likely approach to implement genetically modified mosquitoes in malaria control is the introduction and spread of refractoriness genes in wild mosquito populations [3,4]. A major concern however regarding the spread of refractoriness genes is the possibility that they cannot be integrated into natural malaria vector populations because of gene flow barriers [5] and/or putative genetic adaptation to the environment [6]. Therefore, when developing target control methods, the structure of vector populations, the force of ecological associations and the resulting plasticity of the vectors to local environmental conditions should be considered.

The distributions of mosquito species are related to climate, and in West Africa, it appears that the different chromosomal forms of *An. gambiae s.s*. (Mopti, Bamako, Savanna, Forest and Bissau) occur sympatrically but are segregated environmentally [7-9]. In West Africa published data were compiled to demonstrate that climate variables can be used to map the distribution of *An. gambiae s.s *chromosomal forms [10]. Similar studies have been carried out in Kenya [11] and Nigeria [12]. In addition to climate, anthropogenic environmental alterations such as rice cultivation and irrigation may also affect species composition [13].

In Mali, the *An. gambiae *complex is composed of *An. arabiensis*, and *An. gambiae s.s *Three chromosomal (Mopti, Bamako, Savanna) and two molecular (M and S) forms of *An. gambiae s.s*. have been described and coexist [8,14-16]. The S-molecular form comprises Bamako and Savanna chromosomal forms. A map of their relative frequencies has been produced for a number of specific locations in Mali [15]. Analysis of mosquito data from 16 sites throughout Mali showed a significant negative association between rainfall and the distribution of the Mopti chromosomal form [17]. Variation in the seasonal abundance and infection rates among chromosomal forms of *An gambiae s.s*. in Mali was also observed [18].

The ecological distribution of each chromosomal form seems to be related to a particular epidemiological pattern of the disease. The knock down resistance (*kdr*) allele in the para sodium channel gene, which confers resistance to pyrethroid insecticides, is found in the S-molecular form, but could not be detected in the M-molecular form populations from the same localities [19]. Therefore producing a continuous map of the spatial distribution of their relative frequencies in relation to climate and environmental factors may be useful for conventional and prospective genetically manipulated vector control methods. In this study, published and unpublished vector data were compiled to assess the relationship between the relative frequencies of the different chromosomal forms of *An. gambiae s.s*. with climate and environmental factors, and to produce continuous maps of their spatial distribution.

## Methods

### Description of the study area

The study area covered most of the territory of Mali in West Africa, between 10 and 25° latitude North and 12° longitude West and 4° longitude East. The Country has an area of 1,240,000 square kilometers and an estimated population (United Nations, 2003) of 13,000,000 inhabitants. It is drained by two major rivers (Senegal and Niger) and has 4 distinct eco-climatic zones: i) Southern Sudan savanna with an annual rainfall of 1300–1500 mm from May to October and mean annual thermal amplitude (difference between the mean maximum and the mean minimum temperature) of 5 to 6°C; ii) Northern Sudan savanna with about 700–1300 mm annual rainfall distributed over 4 to 5 months; iii) Sahelian zones with 200–700 mm of annual rainfall distributed over three months and mean annual thermal amplitude of about 12°C; iv) Sub-Sahara zone with less than 200 mm of annual rain and 16°C of annual average thermal amplitude.

Mali is a relatively flat country, altitudinal variations are minimal, ranging from 200 to 350 m above sea level. There are two main seasons varying in length according to latitude: a dry season (November–April) and a rainy season (May–October) characterized by lower temperatures and an increase in humidity. Except for the Sahara desert, the country is entirely endemic for malaria (hyperendemic to hypoendemic from South to North). The main malaria vectors are *An. gambiae s.l*. and *An. funestus*. *An. gambiae s.l*. is composed of *An. arabiensis *and three chromosomal forms of *An. gambiae s.s *named Bamako, Mopti and Savanna [20] and two molecular (M and S) forms [21].

### Data sources and description

#### Vector data

All available published [15] and unpublished data on chromosomal forms of *An. gambiae s.s*. in Mali were collated from cross-sectional and longitudinal surveys carried out between 1981 and 2004 by the Malaria Research and Training Centre (MRTC), University of Bamako, Mali. Most surveys were conducted during the wet season (June–October). Survey sites were mainly small human settlements from 79 distinct rural sites representing various eco-climatic zones of Mali. Because of small distances separating some collection sites, data were aggregated resulting in a set of 71 locations. The database included data collected on i) the total number of *An. gambiae s.s*. specimens, ii) the count of chromosomal (Mopti, Bamako, Savanna and their hybrids/recombinants) forms, and iii) the survey period (month and year). Mosquitoes were collected and processed across surveys following a standardized method to ensure data consistency. Identification of chromosomal forms was by cytogenetic method [22,23].

#### Climatic and environmental data

The climatic and environmental variables which were used in this study included temperature, rainfall, normalized difference vegetation index (NDVI), distance to water bodies, soil water storage (SWS), land use, agro-ecological zones (AEZ) and suitability for malaria transmission. The last one is a binary variable defined from environmental factors related to malaria transmission with cut-off values [24]. The data sources and spatial resolution are the same as described in previous work [25].

For each location, temperature and rainfall data were available as monthly long term averages. NDVI data were also summarized by monthly long term averages of the original decadal values during the period between 1985 and 1995. The agro-ecological zones (AEZ) were distinguished on the basis of the length of the crop growing period and were defined as follow: Equatorial Forest zone (> 270 days), Guinea savanna zone (165 – 270 days), Sudan savanna zone (90 – 165 days) and the Sahelian zone (< 90 days). In Mali only the last three AEZ are found.

### Data analysis

Bivariate multinomial regression models were fitted in STATA 9.0 (STATA Corporation, USA) to assess the association between the relative frequencies of chromosomal forms of *An. gambiae s.s *with climatic and environmental factors. The multinomial outcome data represent the following four chromosomal forms: Mopti, Bamako, Savanna, and others (hybrids Bamako-Savanna and Savanna-Mopti). The Mopti form was considered as the baseline category. The mosquito data obtained at a specific location were linked to the environmental and climate data by drawing a buffer of 2 km around each location and calculating the environmental value by the average of environmental values of all pixels in this buffer.

To take into account the possible lag time between the rainfall and NDVI with the mosquito abundance [26], four summary measures were calculated for each of the two climatic conditions: i) the climatic value during the month of collection (concurrent), ii) the climatic value during the previous month (lag one month), iii) the mean (or total) climatic value during the month of collection and the previous month (2 months average) and iv) the mean (or total) climatic value during the collection month and the two previous months (3 months average). The mean was used as a summary measure for NDVI and the total was considered as a summary measure for rainfall. Vector data obtained from surveys extended over a period longer than a month were available cumulatively for the whole period instead of monthly. In this case the midpoint month was used to relate the climatic factors. The Akaike's Information Criterion (AIC) was used to select the best summary measure and lag time for the rainfall and NDVI. The statistical significance of the environmental factors was assessed using the likelihood ratio test (LRT). All factors with a 15% significance level were entered in a Bayesian geostatistical multinomial regression model. The model took into account spatial heterogeneity by including location-specific random effects at the level of sampling location for each multinomial category (except the baseline). Bayesian kriging was used to assess the spatial patterns of the different chromosomal forms. A description of the geostatistical model is given in the Additional file 1.

## Results

Twenty six thousand three hundred twenty eight mosquitoes (26328) were assigned to one of the 3 chromosomal forms: Mopti, Bamako, and Savanna that represented 57.1%, 19.0% and 18.6% of the chromosomally identified mosquitoes, respectively. The remaining 5.3% were hybrids of Mopti-Savanna or Savanna-Bamako and the recombinants (Table 1). The three eco-climatic zones were sympatric areas for at least 2 of the chromosomal forms. Mopti form was the most abundant, prevailing in all eco-climatic areas with an increasing frequency from South to North (from 51.8% to 95.3%). The opposite situation was observed with Savanna form (1.8% to 25% from North to South). Bamako form was absent in the Sahelian zone. The highest frequency (6.3%) of hybrids/recombinants was observed in the North Sudan savanna.

**Table 1:** Relative frequencies of *An. gambiae s.s. *chromosomal forms by eco-climatic zone in Mali, West Africa.

		**Chromosomal forms of *An. gambiae s.s.***				
**Eco-climatic zones**	**Number of localities**	**Bamako form**	**Mopti form**	**Savanna form**	**Hybrids/Recombinants**	**Total**
Southern Sudan Savanna	10	934 (20.4%)	2375 (51.8%)	1181 (25.8%)	91 (2.0%)	4581
Northern Sudan savanna	33	4060 (20.4%)	10907 (54.8%)	3693 (18.6%)	1248 (6.3%)	19908
Sahelian	36	0 (0.0%)	1752 (95.3%)	33 (1.8%)	54 (2.9%)	1839
Overall	79	4994 (19.0%)	15034 (57.1%)	4907 (18.6%)	1393 (5.3%)	26328

Table 2 presents the results of the bivariate multinomial regression analyses between the chromosomal forms and the environmental and climatic factors used in the analysis. Among the four NDVI and rainfall measures considered in the study, the ones which fitted the distribution of chromosomal forms best (giving smaller AIC) were NDVI mean value and total rainfall value during the month of mosquito collection and the 2 previous months respectively. The results indicate a positive association of the suitability for transmission, the climatic values of NDVI and rainfall (Measure_4) and the SWS index with the relative frequencies of Savanna, Bamako and the hybrids/recombinants chromosomal forms, relative to the Mopti form used as baseline. The Bamako chromosomal form was positively associated with distances of 4–10 km to water bodies and crop/grass/mosaic land use categories, while the hybrids/recombinants chromosomal form was positively associated with Guinea savanna AEZ. All other parameters or category of parameters included in the analysis were negatively associated with Savanna, Bamako and hybrids/recombinants chromosomal forms except distance of >10 – 20 km to water bodies with Savanna form were not significant.

**Table 2:** Bivariate association between chromosomal forms and climate and environmental parameters arising from multinomial regression model.

**Parameters**	**Savanna OR (95% CI)**	**Bamako OR (95% CI)**	**Hybrids OR (95% CI)**	**p-value (AIC)**
**Agro-ecological zones (AEZ)**				
Guinea savanna	1.00	1.00	1.00	< 0.001
Sudan savanna	0.91 (0.84–0.98)	0.87 (0.80–0.94)	1.42 (1.23–1.63)	
Sahel	0.30 (0.26–0.33)	1.03 (0.94–1.12)	0.73 (0.61–0.88)	
**Distance to water bodies**				
< 4 km	1.00	1.00	1.00	< 0.001
4 – 10 km	0.59 (0.55–0.63)	1.10 (1.03–1.18)	0.39 (0.34–0.44)	
>10 – 20 km	0.95 (0.87–1.05	0.80 (0.72–0.89)	0.76 (0.65–0.89)	
> 20 km	0.32 (0.25–0.42)	0.22 (0.15–0.30)	0.32 (0.21–0.49)	
**Land use**				
Savanna	1.00	1.00	1.00	< 0.001
Crop/Grass/Mosaic land	0.34 (0.31–0.37)	1.20 (1.12–1.29)	0.60 (0.52–0.69)	
Others	0.09 (0.07–0.13)	0.10 (0.07–0.14)	0.35 (0.25–0.49)	
**Suitability to transmission**				
Not suitable	1.00	1.00	1.00	< 0.001
Suitable	4.44 (4.14–4.76)	1.67 (1.57–1.78)	2.90 (2.60–3.24)	
**Rainfall**				
Measure_1	1.06 (1.03–1.10)	1.00 (0.96–1.03)	1.23 (1.18–1.29)	<0.001 (58461.76)
Measure_2	1.60 (1.55–1.65)	0.99 (0.96–1.03)	1.31 (1.24–1.37)	<0.001 (57481.14)
Measure_3	1.32 (1.28–1.36)	0.99 (0.96–1.03)	1.29 (1.23–1.35)	<0.001 (58134.34)
Measure_4	1.87 (1.81–1.93)	1.07 (1.04–1.11)	1.51 (1.44–1.59)	**<0.001 (56806.59)**
**NDVI**				
Measure_1	2.09 (2.02–2.17)	1.13 (1.09–1.16)	1.71 (1.62–1.81)	<0.001 (56443.11)
Measure_2	2.69 (2.59–2.79)	1.24 (1.20–1.28)	1.73 (1.63–1.83)	<0.001 (55413.54)
Measure_3	2.45 (2.36–2.54)	1.18 (1.15–1.22)	1.75 (1.65–1.85)	<0.001 (55830.97)
Measure_4	2.81 (2.70–2.92)	1.19 (1.15–1.23)	1.80 (1.70–1.91)	**<0.001 (55171.33)**
**Temperature**				
Mean minimum	0.995(0.984–0.987)	0.995(0.994–0.996)	0.992(0.990–0.994)	<0.001
Mean maximum	0.981(0.980–0.983)	0.993(0.992–0.994)	0.985(0.984–0.987)	<0.001
**SWS**	28.79 (23.59–35.14)	2.15 (1.67–2.77)	7.57(5.39–10.63)	<0.001

Odds ratios are relative to Mopti chromosomal form.

The multivariate spatial multinomial regression model showed a positive association between the SWS index and suitability for transmission and negative association between the minimum temperature and all the chromosomal forms (Table 3). In addition, positive association was observed between NDVI and Savanna form, between maximum temperature and Bamako form and between rainfall, maximum temperature and the hybrids/recombinants. Negative association was observed between North savanna, Sahel and Savanna form; between the minimum temperature and Bamako and between distances of 4–20 km to water bodies, AEZ and the hybrids. The SWS index and suitability for transmission were positively associated and the minimum temperature negative associated with all chromosomal forms in both models. The AEZs significantly associated with all chromosomal forms in the bivariate analyses were no longer significant in the spatial model for Bamako form. The maximum temperature for Bamako and the hybrids/recombinants and the rainfall for the hybrids/recombinants remained significant in the spatial analysis. The distance at which correlation between 2 locations was less than 5% was 428.2 km (101.2, 1755.2), 1113.4 km (327.0, 2135.6) and 953.2 km (318.1, 2090.0) for Savanna, Bamako and the hybrids/recombinants chromosomal forms respectively, indicating a large spatial correlation in the data.

**Table 3:** Odds ratios for presence of different chromosomal forms estimated from the geo-statistical Bayesian multiple multinomial regression model.

**Parameters**	**Savanna**	**Bamako**	**Hybrids/Recombinants**
	
	**Posterior median (95%CI)**	**Posterior median (95%CI)**	**Posterior median (95%CI)**
Rainfall	0.95 (0.83–1.08)	1.09 (0.99–1.20)	1.22 (1.03–1.46)
Max temperature	0.74 (0.47–1.07)	6.09 (4.29–7.99)	2.32(1.34–3.97)
Min temperature	0.41(0.22–0.99)	0.07 (0.04–0.14)	0.28 (0.14–0.58)
NDVI	1.46 (1.30–1.65)	1.04 (0.96–1.13)	1.03 (0.88–1.19)
SWS	2.02 (1.42–2.84)	5.98 (4.45–8.04)	3.25 (1.99–5.32)
**Distance to water bodies**			
< 4 km	1.00	1.00	1.00
4 to 10 km	0.20 (0.05–0.89)	1.52 (0.40–7.01)	0.42 (0.17–0.89)
>10 to 20 km	0.94 (0.15–7.88)	1.64 (0.14–14.05)	0.18 (0.04–0.74)
> 20 km	0.69 (0.09–4.49)	3.66(0.22–56.66)	0.31 (0.07–1.33)
**Suitability to transmission**			
Suitable	1.00	1.00	1.00
Not suitable	4.72(3.43–6.63)	24.76 (16.03–37.77)	3.53 (2.34–5.65)
**Agro-ecological zones (AEZ)**			
South savanna	1.00	1.00	1.00
North savanna	0.29 (0.07–2.00)	2.92 (0.31–29.56)	0.24 (0.06–0.82)
Sahel	0.01 (0.00–0.79)	0.00 (0.00–13.85)	0.05 (0.00–0.92)
**Spatial parameters**			
3/ρ ** (km)	428.2 (101.2–1755.2)	1113.4 (327.0–2135.6)	953.2 (318.1–2090.0)
σ^2^	9.95 (4.45–37.00)	24.95 (8.29–67.78)	8.57 (3.47–22.58)

*Odds ratios are relative to Mopti form

**Distance (km) with spatial correlation < 5%

**Figure 1:**
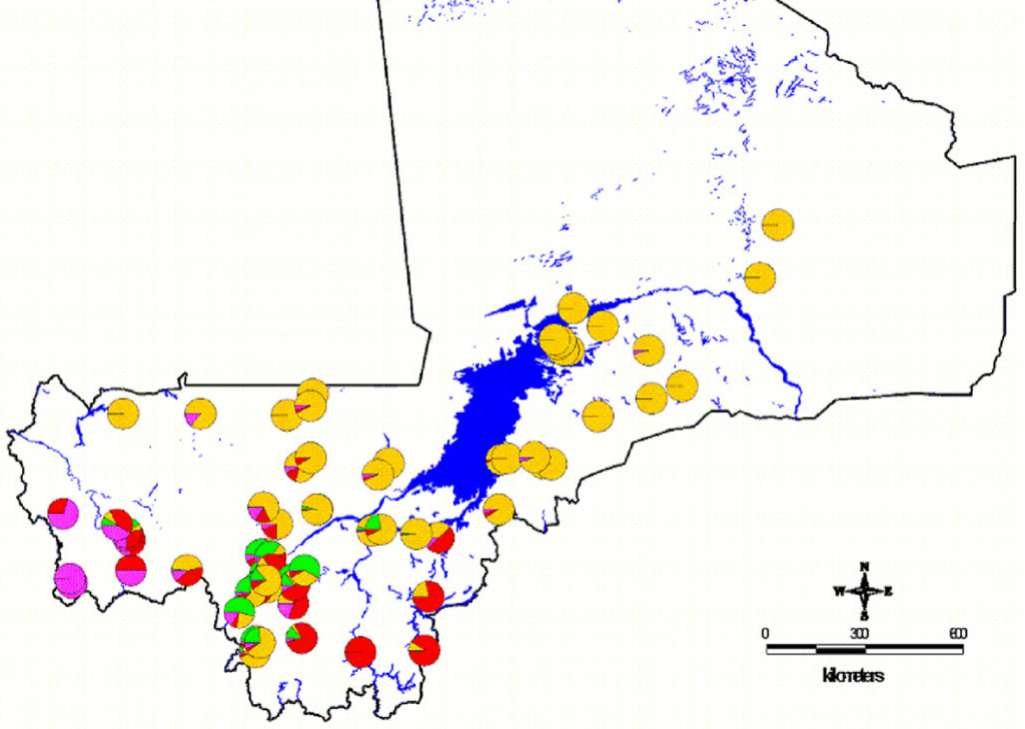
**Observed relative frequencies of the chromosomal forms in 71 locations in Mali, West Africa.** The orange represents Mopti, the red Savanna, the green Bamako and the purple the hybrids/recombinants relative frequencies.

**Figure 2:**
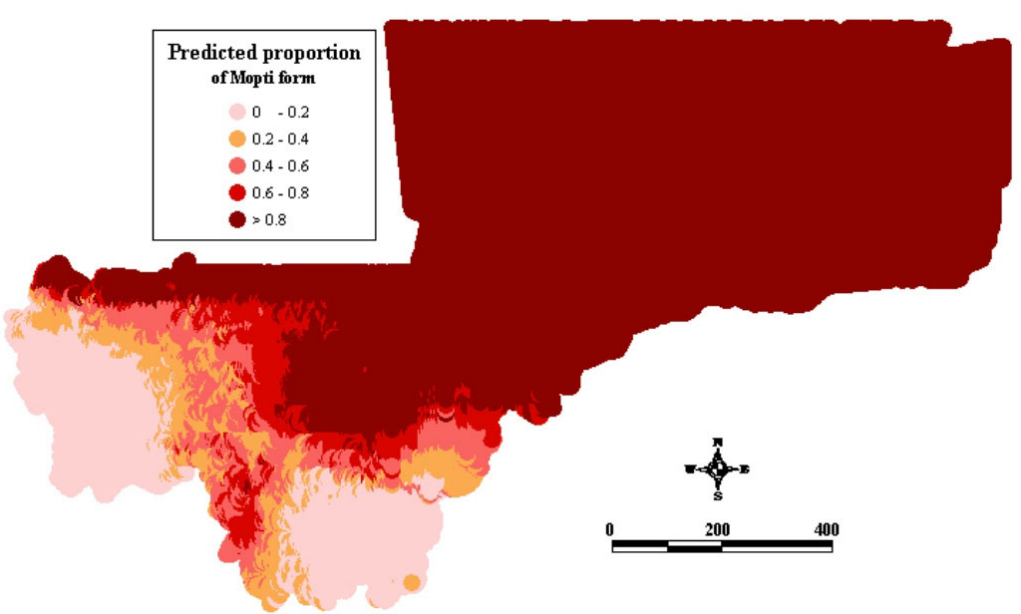
Map of the predicted proportion of the Mopti chromosomal form of *An. gambiae s.s*. in Mali, West Africa.

**Figure 3:**
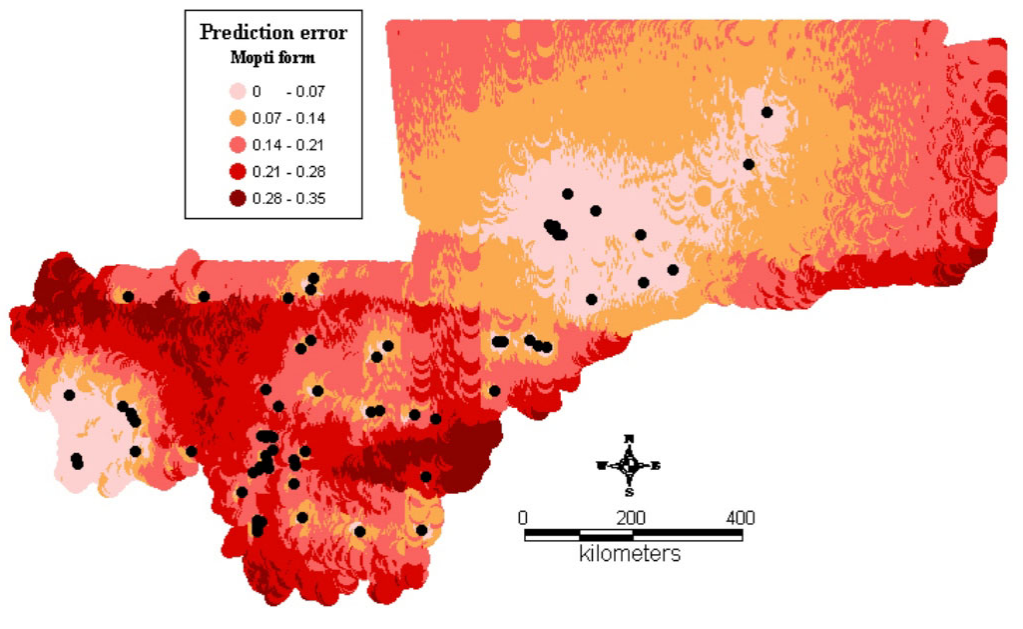
**Map of the prediction errors of the Mopti chromosomal form of *An. gambiae s.s*. in Mali, West Africa.** The black dots represent the data locations.

**Figure 4:**
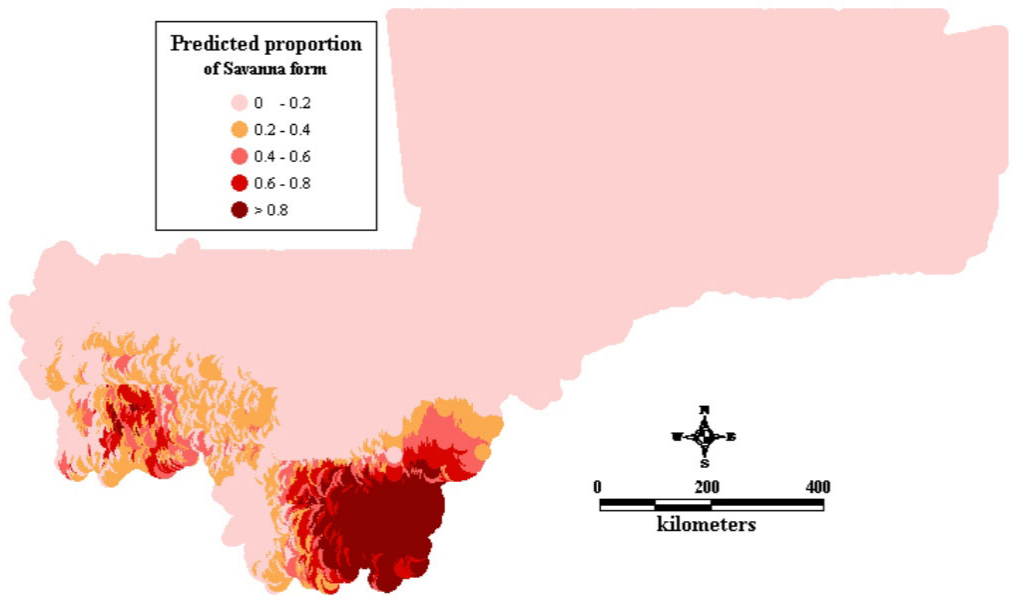
Map of the predicted proportion of the Savanna chromosomal form of *An. gambiae s.s. *in Mali, West Africa.

**Figure 5:**
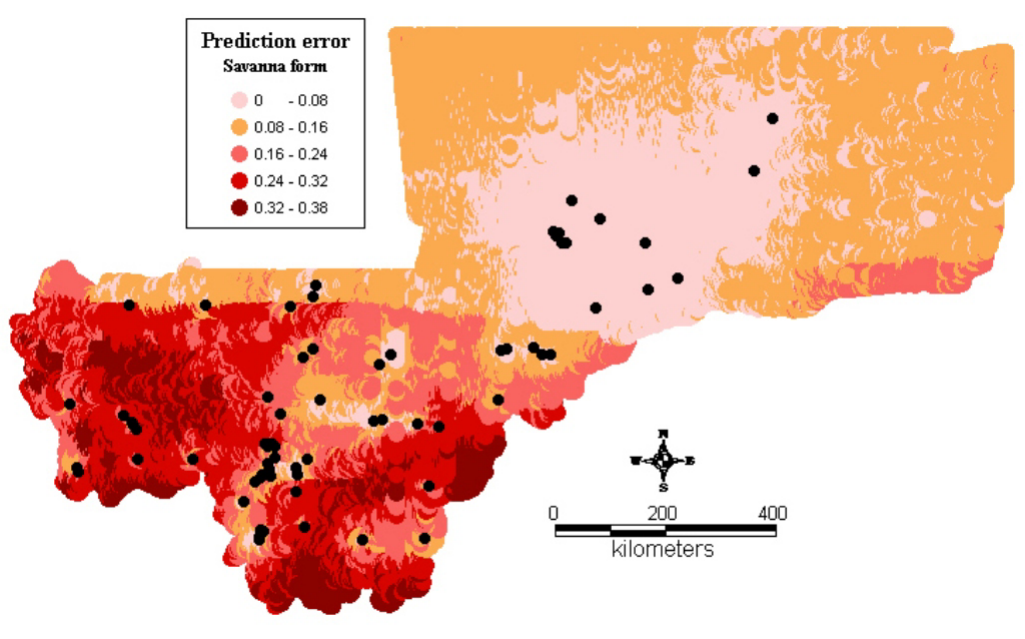
**Map of the prediction errors of the Savanna chromosomal form of *An. gambiae s.s. *in Mali, West Africa.** The black dots represent the data locations.

**Figure 6:**
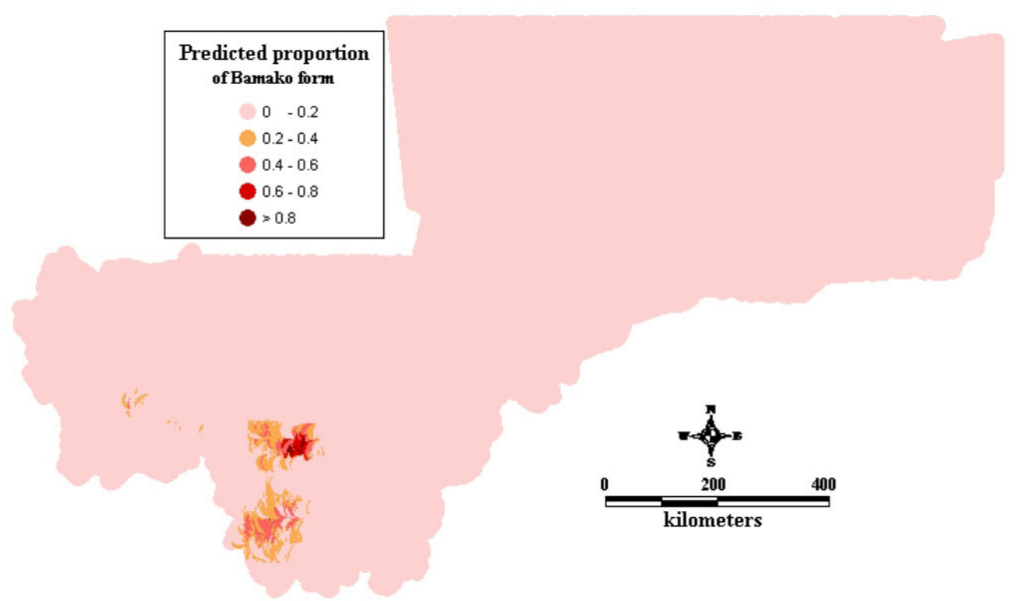
Map of the predicted proportion of the Bamako chromosomal form of *An. gambiae s.s*. in Mali, West Africa.

**Figure 7:**
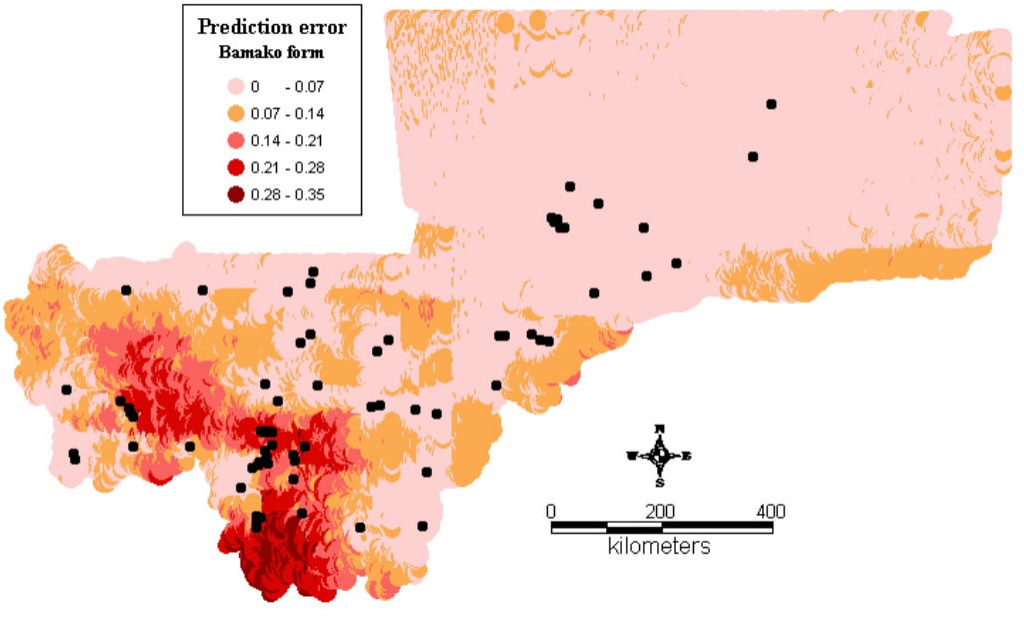
**Map of the prediction errors of the Bamako chromosomal form of *An. gambiae s.s. *in Mali, West Africa.** The black dots represent the data locations.

**Figure 8:**
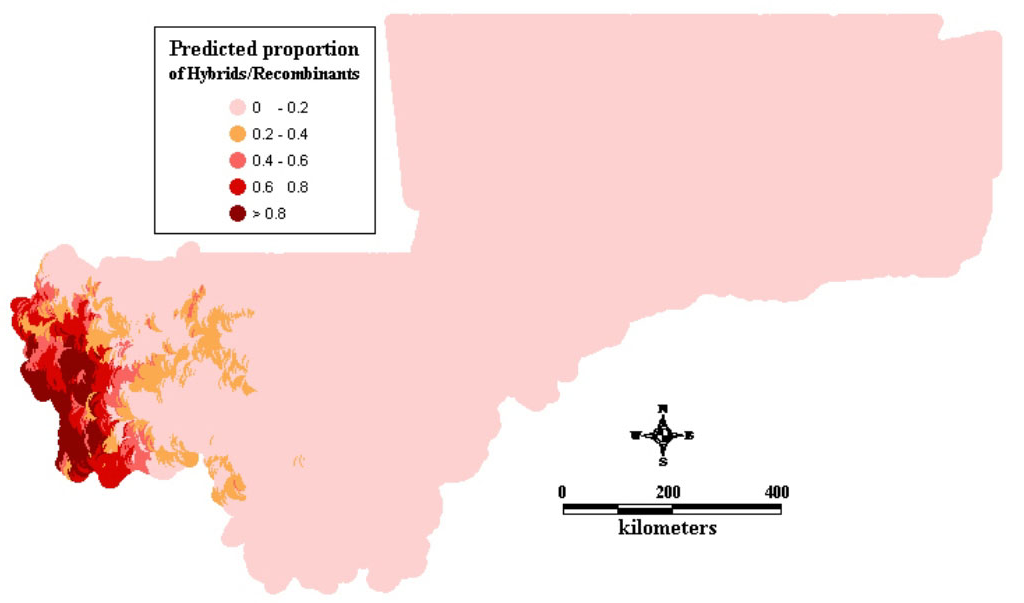
Map of the predicted proportion of the hybrids chromosomal form of *An. gambiae s.s*. in Mali, West Africa.

**Figure 9:**
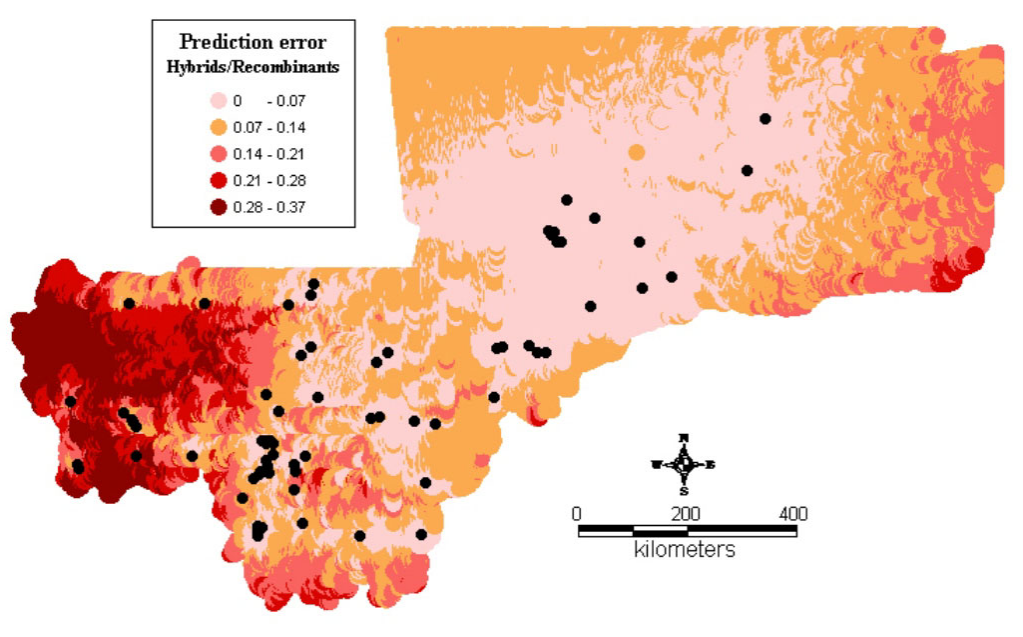
**Map of the prediction errors of the hybrids chromosomal form of *An. gambiae s.s. *in Mali, West Africa. **The black dots represent the data locations.

Figure1 shows the observed relative frequencies of the different chromosomal forms in 71 locations across the country. The spatial distribution maps (Figs 2, 3, 4, 5, 6, 7, 8 and 9) show clearly an ecological aggregation of the different chromosomal forms. The Mopti form (Figs 2, 3) shows a South-North distribution pattern with an increasing frequency reaching up to 100% in the inner delta of Niger River and the Sahelo-Saharian part of the country. The Savanna form (Figs 4, 5) is present in the Sudan savanna area at the South and South-Eastern parts of the region of Kayes and Sikasso respectively. Bamako chromosomal form (Figs 6, 7) is confined to the Western part of the region of Sikasso and the hybrids/recombinants of Bamako-Savanna, Mopti-Savanna (Figs 8, 9) are observed in the South-Western part of the region of Kayes.

## Discussion

The predicted maps of the different chromosomal forms of *An. gamabiae s.s*. represent an average relative frequency over the malaria transmission season in Mali (June to November). They may not reflect the exact situation – which is temporally dynamic – because (i) data were obtained from cross-sectional surveys carried out during a single point of time, and (ii) Long term averages of climatic and environmental factors were used because some of these factors were not available during the survey times. Despite the long duration of the data collection, standardized techniques were used for sampling and processing mosquitoes across surveys rendering the mosquito database consistent.

The analysis of the observed data showed that at least two of the chromosomal forms were sympatric in each of the three eco-climatic zones of Mali. The Mopti chromosomal form was prevalent in all eco-climatic zones indicating that this type can easily adapt to different environmental and climatic conditions. Its chromosomal arrangement bc/bc and u/u may play an important role in its adaptation to diverse environment [15]. Indeed, seasonal variations of the frequency of Mopti chromosomal arrangement show that the frequency of bc karyotype decreases in the rainy season and increases in the dry season, but the frequencies of u karyotype show the reverse variation [17]. The Bamako form which is normally present along river systems, was absent around the Niger River in the Sahelian zone showing the preference of this type to more humid climate. The Savanna form was present in all eco-climatic zones, but with higher frequency in the South Sudan savanna. The three chromosomal forms were sympatric in the Northern Sudan savanna where the highest relative frequencies of the hybrids Mopti-Savanna and Bamako-Savanna were also observed.

The spatial distribution maps clearly show that, in spite of their sympatry, the spatial distribution of the different chromosomal forms is not random. Each chromosomal form favours a particular defined eco-climatic zone as reported by previous studies [7,10,15,27]. The Mopti form (Figs 2, 3) is present country wide but prefers the dryer northern Sahel and the flooded/irrigated areas of the delta of Niger River. Because of it association with flooded plains and irrigated fields, it also breeds continuously even throughout the dry season [15]. The Savanna form (Figs 4, 5) favours the Sudan savanna areas and is particularly predominant in the South and South-Eastern parts of the country (Kayes and Sikasso regions). The Bamako form (Figs 6, 7) has strong preference to specific environmental conditions and it was confined in the Western part of Sikasso region and around Bamako town which also gave the name to this type [14].

The hybrids/recombinants (Figs 8, 9) are observed in the Western part of the country (Kayes region), a wooded area, at the border of the Republic of Guinea Conakry. The spatial distribution of these inversions shows a strong association with ecological/climatic zones [7,27]. The border of the Republic of Guinea Conakry and Kayes is a transitional area between the forest (with high inversion diversity within mosquito populations with more standard and heterozygous carriers) and Savanna (with more homozygous carriers). Field population studies revealed a low frequency of hybrids between Mopti and Savanna and between Bamako and Savanna as well as a complete reproductive isolation between Bamako and Mopti [20]. Therefore, the hybrids/recombinants observed here are likely to be from Bamako-Savanna because these 2 forms are sympatric in this part of the country. It has also be reported that the karyotypes identified as hybrids are in fact not hybrids, but the consequence of low frequency polymorphisms in one or the other taxon [28]. The high spatial correlation observed in the data may probably be due to the effect of environmental factors which influence large areas.

The only spatially-continuous map of *An. gambiae s.s. *chromosomal form distribution produced so far was for West Africa [10]. Our introduced approach, however, yielded a more finely resolved *An. gambiae s.s*. chromosomal form spatially-continuous distribution for Mali. Based on current knowledge on vector resistance to pyrethroids in Mali [19], these maps provide valuable information for selective and targeted malaria vector control in Mali. Indeed, the Mopti chromosomal form – which have not yet developed resistance to insecticide – prevails in the Sahelian and irrigated/flooded areas, while the S molecular form (Savanna and Bamako) – which carries the *kdr *gene – is more abundant in the southern part of the country, particularly in Sikasso and Kayes regions. Although any vector control by means of insecticides must be accompanied by a resistance monitoring system, particular attention must be paid to the southern part of the country.

The maps may also be useful for planning future implementation of malaria control by genetically manipulated mosquitoes. However, more bio-ecological and gene flow studies among the different chromosomal forms are needed before undertaking any field implementation of control by genetically manipulated mosquitoes. In addition, temporal distribution maps of the chromosomal forms would be useful to complete the stratification for targeted vector control. Indeed, in areas where the chromosomal forms occur sympatrically; their relative frequencies change seasonally, most likely in response to annual fluctuations in climate [29]. However, collecting temporal genotyped data is not an easy task because of the skilled and labor intensive techniques required for field identification of the chromosomal forms.

## Conclusion

Our study represents more finely resolved spatially-continuous distribution maps of *An. gambiae s.s*. chromosomal form in Mali. The maps provide valuable information for selective vector control in Mali (insecticide resistance management) and may serve as a decision support tool for the basis for future malaria control strategies including genetically manipulated mosquitoes.

## Competing interests

The authors declare that they have no competing interests.

## Authors' contributions

NS, PV, and MMB conceived the study. PV, LG, and NS performed the statistical analysis. NS has written the ms with input from PV, TS, SD, and SFT. GD and YTT provided vector data. TS and YTT oversee the work. All authors read the final version and approved it.

## Supplementary Material

Additional file 1 Geostatistical multinomial regression model. The data provided represent the formulation of the spatial statistical model and the model fit. Click here for file
