# Supplementary material for: Spatial distribution of the chromosomal forms of anopheles gambiae in Mali
Source: Malar J. 2008 Oct 10;7:205. doi: 10.1186/1475-2875-7-205 (PMC2579919; doi:10.1186/1475-2875-7-205)
Supplement: Additional file 1 — Geostatistical multinomial regression model. The data provided represent the formulation of the spatial statistical model and the model fit. [file 1475-2875-7-205-S1.doc]

## Additional file 1

## Geostatistical multinomial regression model

Let $Y_{ik}$ be the observed frequency of mosquito chromosomal form *k* at location *i* where *k=1,2,3,4* denote the Mopti, Bamako, Savanna , and hybrid forms, respectively. It was assumed that $Y_{ik}$ arise from a multinomial distribution, that is $\left(Y_{i1},Y_{i2},Y_{i3},Y_{i4}\right)\sim Mult\left(n_{i},\pi_{i1},\pi_{i2},\pi_{i3},\pi_{i4}\right)$ with parameters $\pi_{ik}$ and $n_{i}$ is the total number of *An. gambiae s.s* collected at location *i*. Spatial correlation was introduced on the location-specific random effects $\varphi_{ik}$which are modeled together with the covariate effects on the logit parameters, that is $\log\left(\frac{\pi_{ik}}{\pi_{i4}}\right)=\underline{X}_{i}^{T}\underline{\beta}_{k}+\varphi_{ik}$ where $\underline{\beta}_{k}$are covariate parameters related to the *kth* multinomial category, *k=1,2,3*.

It was also assumed $\varphi_{ik}$ to model a latent isotropic Gaussian spatial process, that is$\varphi_{k}=(\varphi_{1k},...\varphi_{Nk})\sim MVN(0,\Sigma_{k})$, with covariance matrix$\Sigma_{k}$ and that spatial correlation between any pair of locations is a function of distance between locations, that is $\left(\Sigma_{k}\right)_{ij}=\sigma_{k}^{2}\exp(-\rho_{k}d_{ij})$ where $\sigma_{k}^{2}$is the spatial variance related to the multinomial category k, $\rho_{k}$ is the parameter that models the rate of correlation decay and *d_ij_* the distance between the locations *i* and *j.* Based on the above specification, the minimum distance for which the spatial correlation becomes less than 5% is calculated by [1]. The model parameters were estimated using Markov Chain Monte Carlo (MCMC) simulation methods. Bayesian kriging was used to predict the species frequency at 85,000 unsampled locations [2]. The Bayesian model fit was carried out in WinBUGS 1.4. (Imperial College and MRC, UK), whereas the model prediction was implemented in Fortran 95 (Compaq Visual Fortran, Professional 6.6.0) using standard numerical libraries (NAG, The Numerical Algorithms Group Ltd).

## Model fit

The parameters of the above models were estimated using Markov Chain Monte Carlo (MCMC) simulation methods. In accordance with the Bayesian model specification, prior distributions were adopted for the model parameters. Vague normal prior distributions were chosen for $\beta_{-}$ parameters with large variances (i.e., 10,000), gamma prior for$r$, inverse gamma priors for $\sigma_{k}$and uniform priors for$\rho_{k},k=1,2,3$. A single chain sampler was run with a burn-in of 5,000 iterations. Convergence was assessed by inspection of ergodic averages of selected model parameters. Bayesian kriging was used to predict the species frequency at 85,000 unobserved locations [2]. The Bayesian model fit was carried out in WinBUGS 1.4. (Imperial College and MRC, UK), whereas the model prediction was implemented in Fortran 95 (Compaq Visual Fortran, Professional 6.6.0) using standard numerical libraries (NAG, The Numerical Algorithms Group Ltd).

**References**

1. Ecker M, Gelfand AE: **Bayesian variogram modelling for an isotropic spatial process**. *Journal of Agricultural, Biological and Environmental Statistics* 1997,**4:** 347-369.

2. Diggle PJ, Tawn JA: **Model-based geostatistics**. *Applied statistics* 1998, **47:** 299-350.
